# Bioavailability of Melatonin from Lentil Sprouts and Its Role in the Plasmatic Antioxidant Status in Rats

**DOI:** 10.3390/foods9030330

**Published:** 2020-03-12

**Authors:** Miguel Rebollo-Hernanz, Yolanda Aguilera, Teresa Herrera, L. Tábata Cayuelas, Montserrat Dueñas, Pilar Rodríguez-Rodríguez, David Ramiro-Cortijo, Silvia M. Arribas, María A. Martín-Cabrejas

**Affiliations:** 1 Department of Agricultural Chemistry and Food Science, School of Science, Universidad Autónoma de Madrid, 28049 Madrid, Spain; miguel.rebollo@uam.es (M.R.-H.); yolanda.aguilera@uam.es (Y.A.); 2 Institute of Food Science Research, CIAL (UAM-CSIC), 28049 Madrid, Spain; teresa.herrera@uam.es (T.H.); cial.foodchem@gmail.com (L.T.C.); 3 Grupo de Investigación en Polifenoles, Unidad de Nutrición y Bromatología, Facultad de Farmacia, Universidad de Salamanca, Campus Miguel de Unamuno, 37007 Salamanca, Spain; mduenas@usal.es; 4 Department of Physiology, School of Medicine, Universidad Autónoma de Madrid, 28029 Madrid, Spain; pilar.rodriguezr@uam.es (P.R.-R.); dramiro@bidmc.harvard.edu (D.R.-C.); silvia.arribas@uam.es (S.M.A.)

**Keywords:** melatonin, bioavailability, lentil sprouts, phenolic compounds, antioxidant status, pharmacokinetics

## Abstract

Melatonin is a multifunctional antioxidant neurohormone found in plant foods such as lentil sprouts. We aim to evaluate the effect of lentil sprout intake on the plasmatic levels of melatonin and metabolically related compounds (plasmatic serotonin and urinary 6-sulfatoxymelatonin), total phenolic compounds, and plasmatic antioxidant status, and compare it with synthetic melatonin. The germination of lentils increases the content of melatonin. However, the phenolic content diminished due to the loss of phenolic acids and flavan-3-ols. The flavonol content remained unaltered, being the main phenolic family in lentil sprouts, primarily composed of kaempferol glycosides. Sprague Dawley rats were used to investigate the pharmacokinetic profile of melatonin after oral administration of a lentil sprout extract and to evaluate plasma and urine melatonin and related biomarkers and antioxidant capacity. Melatonin showed maximum concentration (45.4 pg/mL) 90 min after lentil sprout administration. The plasmatic melatonin levels increased after lentil sprout intake (70%, *p* < 0.05) with respect to the control, 1.2-fold more than after synthetic melatonin ingestion. These increments correlated with urinary 6-sulfatoxymelatonin content (*p* < 0.05), a key biomarker of plasmatic melatonin. Nonetheless, the phenolic compound content did not exhibit any significant variation. Plasmatic antioxidant status increased in the antioxidant capacity upon both lentil sprout and synthetic melatonin administration. For the first time, we investigated the bioavailability of melatonin from lentil sprouts and its role in plasmatic antioxidant status. We concluded that their intake could increase melatonin plasmatic concentration and attenuate plasmatic oxidative stress.

## 1. Introduction

Melatonin (*N*-acetyl-5-methoxytryptamine) is a multifunctional indoleamine acting as a neurohormone and bioactive antioxidant synthesized from L-tryptophan via serotonin in both plants and animals [1]. Melatonin is considered an excellent naturally occurring antioxidant, displaying free radical and reactive species scavenging properties. Melatonin has been shown to detoxify different (i) reactive oxygen species (ROS), e.g., singlet oxygen, hydrogen peroxide, and hydroxyl radicals; reactive nitrogen species (RNS), e.g., peroxynitrite, and peroxynitrous acid, and nitric oxide; and (iii) reactive chlorine species (RCS), such as hypochlorous acid [2]. Additionally, melatonin upregulates the expression and activity of several antioxidant enzymes, preserving the optimal function of mitochondria, and contributing to the attenuation of oxidative stress [3]. Melatonin may be associated with the prevention of chronic diseases related to aging and oxidative stress, including type-2 diabetes, obesity, and cardiovascular diseases [4,5,6,7].

Besides the significant influence of the light-dark cycle, nutritional factors such as macro- and micro- nutrient intake, meal timing, and type of diet can also modulate the concentration of melatonin in plasma [8]. The inclusion of melatonin-rich plant foods in the diet may promote human health owing to its biological activities and bioavailability [9,10]. Melatonin is broadly used as a food supplement worldwide, including in the United States and Europe, to counteract physiologically diminished activity of the pineal gland [11]. The European Food Safety Authority (EFSA) has established guidance on regulating melatonin as a dietary supplement, unlike the U.S. Food and Drug Administration (FDA). The EFSA only supports melatonin administration for the treatment of sleeping disorders and jet lag. Moreover, certain European countries have restricted melatonin use to decreasing quantities [12]. Hence, the search for novel natural food sources of melatonin is the object of increasing interest. Melatonin-rich food consumption could influence health by increasing the plasmatic melatonin levels and improving antioxidant status [13].

Melatonin has been found in noteworthy concentrations in teas and herbal infusions [14], and other foodstuffs such as grapes [15], olive oil, cereals, fruits, nuts [16], and legumes [17]. Fermentation food processes increase melatonin concentration, as shown in beer and wine, among others [18]. Germination has been commonly used for the enhancement of legume nutritional value. This processing significantly diminishes the concentration of nonnutritional compounds, enhances the digestibility of proteins, and modifies dietary fiber fractions. Furthermore, germination magnifies the content of bioactive antioxidant compounds, including melatonin [19,20,21].

The bioavailability of plant-food-derived melatonin has been confirmed, with reported increments in plasmatic melatonin levels upon consumption of enriched melatonin foods [22]. Synthetic melatonin is absorbed in the gastrointestinal tract 45 min post-oral administration, following metabolism in the liver. From 3 to 76% of melatonin consumed reaches the circulation, and it has a short half-life, *t_1/2_* (i.e., about 20−40 min) [23]. Then, melatonin is mainly excreted as sulfated metabolites in the urine, primarily as 6-sulfatoxymelatonin (aMT6s) [24]. Research on the bioavailability and pharmacokinetics of dietary melatonin is quite scarce to date. Despite the influence of the food matrix and digestion processes on the absorption of this amphipathic molecule, studies have revealed that the intake of melatonin-rich foods gives rise to an increase in circulating melatonin concentrations [25,26].

Lentils are a source of bioactive compounds, including dietary fiber, protease inhibitors, lectins, saponins, phytosterols, and phenolic compounds. Germination generally increases the content of soluble components. Minor compounds found in lentils and lentil sprouts are considered to be responsible for their health-promoting effects [27]. Lentils have been shown to possess a wide range of biological activities demonstrated not only *in vitro*, but also *in vivo* and by clinical trials; antioxidant, antidiabetic, antiobesity, cardioprotective, and cancer-preventive are the most remarkable properties [28].

Therefore, we aimed to evaluate the influence of the intake of lentil sprouts on the plasmatic concentration of melatonin and metabolically related compounds (plasmatic serotonin and urinary 6-sulfatoxymelatonin), as well as total phenolic compounds and the effects on plasmatic antioxidant status in comparison to synthetic melatonin administration.

## 2. Materials and Methods

### 2.1. Materials

Synthetic melatonin (> 99%), Folin-Ciocalteu reagent, gallic acid (> 96%), Na_2_CO_3_, 3′,6′-dihydroxyspiro[isobenzofuran-1(3H),9′-[9H]xanthen]-3-one (fluorescein), 2,2-azobis (2-methyl-propionamidine)-dihydrochloride, 6-hydroxy-2,5,7,8-tetramethylchroman-2-carboxylic acid (Trolox), 2,4,6-tri(2-pyridyl)-s-triazine (TPTZ), and FeCl_3_ were obtained from Sigma-Aldrich (St. Louis, MO, USA). All other chemicals and reagents were obtained from VWR (Barcelona, Spain) unless otherwise specified. Melatonin, 6-sulfatoxymelatonin, and serotonin ELISA kits were obtained from IBL-International (Hamburg, Germany). Male Sprague-Dawley rats (3 weeks old) were acquired at the Animal House Facility of the Universidad Autónoma de Madrid (Madrid, Spain). Diet was purchased from Safe (Augy, France). Raw lentils (*Lens culinaris* L., var. Castellana) were provided by the Institute of Food Science, Technology and Nutrition (ICTAN-CSIC, Madrid).

### 2.2. Lentil Sprout Extracts

**Figure 1:**
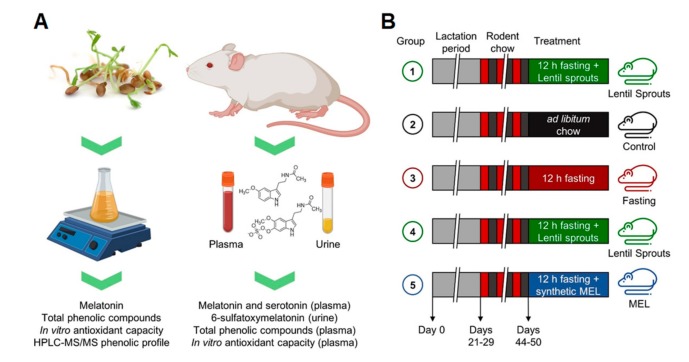
Schematic diagram of the experiments design of this research: (**A**) Lentils were germinated for six days, and phytochemicals (melatonin and phenolic compounds) were extracted and characterized, as was the *in vitro* antioxidant capacity of the product. Lentil sprouts were given to rats and plasma and urine were used for the measurement of melatonin, serotonin, 6-sulfatoxymelatonin (immunochemically), and phenolic compounds and the *in vitro* antioxidant capacity following the protocol here illustrated: (**B**) The first group of rats fasted for 12 h. They were then administered a lentil sprout extract via gavage followed sequential blood extraction for the pharmacokinetic study. Groups 2–5 were used in the bioavailability/bioactivity study and subjected to different feeding conditions: ad libitum standard rat chow (Control), 12 h fasting (Fasting), Lentil sprout ingestion after a 12 h fasting (Lentil Sprouts), and the administration of a melatonin solution (MEL) after 12 h fasting, followed in all the cases by blood and urine sampling.

Lentils were germinated following the procedure described in Aguilera et al. [29]. This process presented good viability, with 96% germination. Lentil sprouts were frozen, freeze-dried, ground, packed in plastic bags, and stored at −20 °C. The analysis of sprouts was carried out in triplicate to determine the melatonin and phenolic compound contents, as well as the *in vitro* antioxidant capacity, as described previously [29]. Melatonin was measured using HPLC-ESI-MS/MS, whereas total phenolic compounds were evaluated using the Folin-Ciocalteu method, and *in vitro* antioxidant capacity was measured by the ORAC method, using spectrophotometric methods; the phenolic profile was characterized suing HPLC-DAD-MS/MS techniques (Figure 1A). The extract from lentil sprouts was prepared by mixing lentil sprout flour (20 g) with ethanol (150 mL) and shaking for 16 h at 4 °C in darkness. The mixture was sonicated for 15 min and filtered with vacuum through 11 µm filters (Whatman). The extract was evaporated at 30 °C to dryness and dissolved in 3 mL phosphate-buffered saline (PBS) buffer to achieve the dose. A synthetic melatonin solution was prepared in PBS to deliver the same dose of the lentil sprouts extract.

### 2.3. Extraction and Analysis of Phenolic Compounds in Raw Lentils and Lentil Sprouts

#### 2.3.1. Extraction of Free and Bound Phenolic Compounds

For free phenolic compound extraction, freeze-dried samples (1 g) were macerated in methanol: HCL (1‰)–water 80:20 (*v/v*) at 4 °C for 16 h. Subsequently, they were centrifuged at 4000 g and 4 °C for 20 min. The extraction process was performed twice. The extracts were combined and concentrated at 30 °C under a vacuum for methanol evaporation. For the extraction of bound phenolics, the residues from the above free phenolic compounds extraction were flushed with N_2_ and hydrolyzed directly with 10 mL of 4 M NaOH at room temperature for 1 h with shaking. The mixture was acidified to pH 2 with concentrated HCl (8M), centrifuged at 4000 g and 4 °C for 20 min, and extracted three times with 1:1 ethyl ether: ethyl acetate. The organic fractions were evaporated to dryness at 30 °C under a vacuum. For phenolic analysis, the dry extracts were dissolved in 10 mL of water. For purification, an aliquot (4 mL) was passed through a C18 solid-phase extraction (SPE) cartridge (Waters, Milford, MA, USA), and phenolic compounds were eluted with methanol. Afterward, extracts were concentrated under a vacuum in a rotary evaporator (30 °C) and then dissolved in aqueous 0.1% TFA:acetonitrile (90:10 v/v) for phenolic compound analysis. The total content of phenolic compounds was obtained as the sum of free and bound phenolics.

#### 2.3.2. HPLC–DAD–ESI/MS^n^ qualitative and quantitative analyses of phenolic compounds

Samples were analyzed using a Hewlett-Packard 1100MS (Agilent Technologies, Palo Alto, CA, USA) chromatograph equipped with a quaternary pump, and a diode array detector (DAD) coupled to an HP Chem Station (rev. A.0504) data-processing station. Solvents used were 0.1% formic acid in water (solvent A) and 100% acetonitrile (solvent B). The elution gradient established was 15% B for 5 min, 15–20% B for 5 min, 20–25% B for 10 min, 25–35% B for 10 min, 35–50% B for 10 min, and re-equilibration of the column. The separation of phenolic compounds was performed in a Spherisorb S3 ODS-2 C8 column (Waters, Milford, USA) (3 μm, 150 mm × 4.6 mm i.d.) operating at 35 °C and a flow rate of 0.5 mL/min. Double online detection was carried out in the DAD using 280 nm and 370 nm as preferred wavelengths. A mass spectrometer (MS) connected to the HPLC system via the DAD cell outlet was used and detection was performed in an API 3200 Qtrap (Applied Biosystems, Darmstadt, Germany) equipped with an ESI source, triple quadrupole-ion trap mass analyzer and controlled by the Analyst 5.1 software. The setting parameters were zero grade air as the nebulizer gas (30 psi), turbo gas for solvent drying (400 °C, 40 psi), and nitrogen served as the curtain (20 psi) and collision gas (medium). The quadrupoles were set at unit resolution. The ion spray voltage was set at −4500 V in the negative mode. The MS detector was programmed to perform a series of two consecutive modes: enhanced MS (EMS) and enhanced product ion (EPI) analysis. EMS was employed to show full scan spectra to give an overview of all the ions in the sample. The settings used were declustering potential (DP) −45 V, entrance potential (EP) −6 V, and collision energy (CE) −10 V.

Spectra were recorded in negative ion mode between *m/z* 100 and 1000. EPI mode was further performed to obtain the fragmentation pattern of the parent ion(s) of the previous experiment using the following parameters: DP: −50 V, EP: −6 V, CE: −25 V, and collision energy spread (CES) 0 V. The phenolic compounds were characterized according to their UV and mass spectra and retention times, and comparison with authentic standards when available. For quantitative analyses, calibration curves were prepared by injection of known concentrations of different standard compounds.

### 2.4. Animals Care and Diet

Experiments were performed in Sprague Dawley rats. All experimental procedures were approved by the Ethics Review Board of Universidad Autónoma de Madrid and conformed to the Guidelines for the Care and Use of Laboratory Animals (NIH publication No. 85–23, revised in 1996), the Spanish legislation (RD 1201/2005) and the Directive 2010/63/EU on the protection of animals used for scientific purposes. The rats were housed under controlled conditions of 22 °C, 40% relative humidity, and 12/12 h light/dark photoperiod. After weaning (day 21–29), rats were kept for 20−25 days under a reversed light/dark cycle (Figure 1; Table 1). They were fed ad libitum with a breeding diet (SAFE A03) containing 51.7% carbohydrates, 21.4% protein, 5.1% lipids, 3.9% fiber, 5.7% minerals, and 12.2% humidity. Drinking water was sterilized by UV, mechanical, and chemical treatments, and provided ad libitum in all cases. All the animals were housed in buckets on aspen wood bedding, which was replaced once a week. The health and welfare of the animals were monitored by staff at least once a day. Monitoring of animals’ health ensured that they were free of any pathogens that may interact with any of the parameters studied.

### 2.5. Pharmacokinetic Study

In a pharmacokinetic study, rats (*n* = 8) were orally administered with a single dose (50 µg melatonin/kg) of lentil sprouts extract via gastric gavage. Animals were previously fasted for 12 h but had free access to water before the experiment. Blood samples were withdrawn at 0, 60, 90, 120, 180, and 240 min after dosing through sublingual bleeding and transferred to vials containing 5% heparin, centrifuged at 4 °C for 15 min at 3000 *g*. Pharmacokinetic parameters were calculated using the PKSolver [30]. The area under the curve (AUC) is the integral of the plasma concentration of an altered drug against an interval of definite time. Pharmacokinetic parameters including plasma elimination rate constant (*K_e_*), elimination half-life (*t_1/2_*), time to reach measured maximum plasma concentration (*T_max_*), measured maximum plasma concentration (*C_max_*), area under the plasma concentration-time curve from the time zero to the time of last quantifiable concentration (AUC_0–t_), area under the plasma concentration-time curve from time zero to infinity (AUC_0–∞_), area under the plasma (first) moment concentration-time curve from time zero to infinity (AUMC_0–∞_), mean residence time (MRT), volume of distribution (*V_D_/F*), and clearance (*Cl/F*) were determined by noncompartmental methods.

### 2.6. Bioavailability and Bioactivity Study

To measure the bioavailability and biological activity of melatonin from lentil sprouts, rats were grown as previously described until day 44–50. At the end of this period, the rats were subjected to four different feeding conditions (*n* = 10 per group): standard rat chow without extract (Control), a 12 h-fasting period without extract (Fasting), a 12 h fasting period followed by administration of lentil sprout extract (50 µg melatonin/kg) (Lentil Sprouts), or a 12 h fasting period followed by the administration of 50 µg synthetic melatonin/kg solution (MEL). Each of the four groups (10 rats) was administered the treatment and sacrificed on a different day. Weights were similar on the day of treatments. Having a small number of rats, we were able to diminish the influence of rat photoperiod (changes in melatonin content due to light cycles). The administration was performed through a gavage using a suitable intubation cannula. After that, the rats were individually caged, and a cling film was placed below the cage to obtain the urine samples. After 90 min of extract administration, all rat groups were anesthetized by CO_2_. Urine was collected from the cling film with a pipette and transferred to a vial. All experiments were carried out at 11 a.m., which was the peak time of melatonin production under the light/dark cycle. The blood was collected by cardiac puncture, transferred to vials containing 5% heparin, and centrifuged at 4 °C for 15 min at 2100 g. The plasma was then divided into aliquots into 1 mL vials and kept frozen at −80 °C to assess several biomarkers related to melatonin metabolism. Before the analysis of melatonin, serotonin, aMT6s, and total phenolic compounds, samples were defrosted on ice in the dark. Phytochemicals and metabolites were isolated using SPE. First, plasma and urine samples were centrifuged at 15000 *g* for 4 min at 4 °C. Then, samples were filtered using C18 SPE cartridges (Waters, Cerdanyola del Vallès, Spain). Finally, the eluted obtained fractions were evaporated to dryness (Speed Vac SC 200, Savant Instruments, Farmingdale, NY, USA) and reconstituted before being assayed. According to the volume obtained, the analyses were carefully designed to maximize the use of biological samples; aliquots were employed to measure melatonin, 6-sulfatoxymelatonin, serotonin, total phenolics, and the *in vitro* antioxidant capacity.

### 2.7. Melatonin Determination

Plasma residues were dissolved in Milli-Q water, and melatonin levels were determined by a competitive enzyme immunoassay kit (Catalog No. RE54021, IBL) according to the manufacturer’s guidelines. The kit is characterized by an analytical sensitivity of 1.6 pg/mL and high analytical specificity (low cross-reactivity).

### 2.8. 6-Sulfatoxymelatonin (aMT6s) Determination

Urine residues were dissolved in tris-buffered saline (TBS) and protected from direct sunlight. aMT6s levels were determined by a competitive enzyme immunoassay kit (Catalog No. RE54031, IBL) according to the manufacturer´s instructions. The assay sensitivity was 1.0 ng/mL.

### 2.9. Serotonin Quantification

Plasma residues were dissolved in Milli-Q water, and serotonin levels were determined by a competitive enzyme immunoassay kit (Catalog No. RE59121, IBL) according to the manufacturer´s guidelines. The kit is characterized by an analytical sensitivity of 2.68 ng/mL and high analytical specificity (low cross-reactivity).

### 2.10. Total Phenolic Compounds (TPC)

Plasma residues were dissolved in Milli-Q water, and total phenolic compounds were determined by Folin-Ciocalteu colorimetric method, according to Singleton et al. [31] using gallic acid as standard. Samples (10 μL) were added to 150 μL of Folin-Ciocalteu reagent (diluted 1:15, *v/v* in Milli-Q water). After exactly 3 min, 20% Na_2_CO_3_ (50 μL) was added to each well. After 120 min at room temperature, the absorbance of the blue complex was measured at 760 nm using a microplate reader. The concentration of total phenolic compounds was expressed as mg GAE/mL.

### 2.11. ORAC (Oxygen Radical Absorbance Capacity)

The above plasma samples were used to determine the radical scavenging activity via the ORAC method using fluorescein as a fluorescence probe [14]. The assay was performed at 37 °C in 75 mM phosphate buffer (pH 7.4). The reaction mixture (200 μL) contained fluorescein (70 nM), 2,2-azobis (2-methyl-propionamidine)-dihydrochloride (12 mM), and samples (Trolox or samples). Fluorescence was read at 485/520 nm, excitation/emission, respectively. Black 96-well untreated microplates (PS Balck, Porvair, Leatherhead, UK) were used. The fluorescence signal was measured every minute for 80 min after automatic shaking. All reaction mixtures were prepared in triplicate, running them at least three times independently. Fluorescence was normalized to the oxidation control (phosphate buffer) and stability control (no antioxidant). The ORAC values were expressed as mM Trolox equivalents (mM TE) according to the protocol previously described [14].

### 2.12. FRAP (Ferric Reducing Antioxidant Power)

The plasma samples were also used to assess the reducing antioxidant potential using the FRAP assay, as previously described [14]. Briefly, 300 μL of a working FRAP reagent [acetate buffer 0.3 M pH 3.6, 10 mM TPTZ in 40 mM HCl and 20 mM FeCl_3_·6H_2_O (10:1:1) (*v/v/v*)] were tempered to 37 °C, and then 10 μL of plasma samples were added. The absorbance was taken at 593 nm against reagent blank after 10 min. FRAP values were calculated and reported as µM Trolox equivalents (µM TE).

### 2.13. Statistical Analysis

Each sample was analyzed in triplicate. Data were reported as mean ± standard deviation (SD). The data were analyzed using the *T*-test or by one-way analysis of variance (ANOVA) and *post hoc* Tukey test. Relationships between the analyzed parameters were evaluated by computing Pearson linear correlation coefficients setting the level of significance at *p <* 0.05, *p* < 0.01, and *p <* 0.001. The statistical analysis was performed by SPSS 23.0.

## 3. Results and Discussion

### 3.1. Lentil Sprouts are a Source of Melatonin and Antioxidant Phytochemicals

Lentil sprouts germinated for 6 days exhibited high amounts of melatonin (1.01 µg/g) and phenolic compounds (TPC accounted for 3.47 mg/g) (Table 2). The melatonin concentration underwent a drastic increase (more than 2000-fold) compared to that of raw lentils. Germination induced a significant (*p* < 0.05) increment in the content of this indoleamine, which has also been seen in other legumes and sprouted vegetables [17]. The concentration of melatonin in these germinated lentils is considered higher than the content in other plant foods [32]. Melatonin has also been found in outstanding levels in fruits, coffee, and its by-products, or fermented foods like wine and beer [33,34]. Nonetheless, the concentration of phenolic compounds (TPC) exhibited a slight decrease (28%), mainly due to the loss of FPC (30% reduction). The proportion free phenolics:bound phenolics (FPC: BPC) remained unchanged. This reduction has also been reported by other authors, not only in lentils [35], but also in beans or chickpeas [36]. The decrease of free phenolics during germination can be associated with their capacity for scavenging ROS in the plant cell during germination. Likewise, phenoloxidase and peroxidase enzymes, triggered during germination, might cause a diminution in the concentration of free phenolics [37].

Because of the changes produced by germination, the antioxidant capacity of the germinated lentils was higher (3.8-fold) in contrast to the potential of raw lentils. The *in vitro* antioxidant capacity of this product can be considered high in comparison with other legumes or plant-foods [38]. Germination has been shown to be a cost-effective and sustainable strategy to increase the antioxidant capacity of legumes [39]. Therefore, germinated lentils were selected to evaluate melatonin bioavailability and their influence on the plasmatic antioxidant status due to their higher content in melatonin and their higher *in vitro* antioxidant capacity.

#### Germination Decreased the Concentration of Phenolic Acids and Flavan-3-ols, Preserving Flavonols

**Figure 2:**
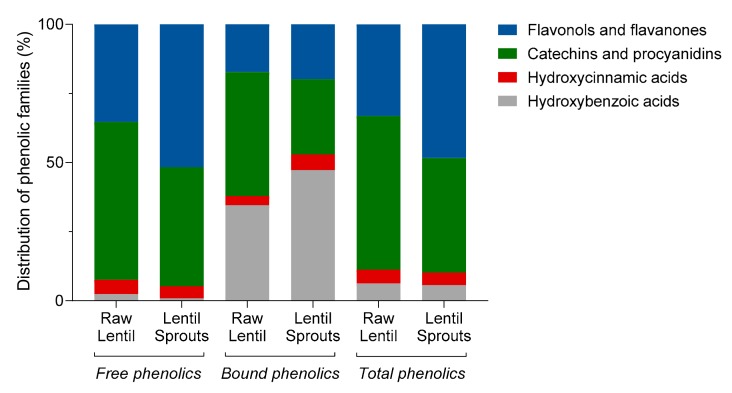
Distribution of different phenolic groups (hydroxybenzoic acids, hydroxycinnamic acids, catechins and procyanidins, and flavonols and flavanones) in the free, bound, and total phenolic fractions of raw and germinated lentils.

A more comprehensive analysis of the antioxidant phytochemicals found in raw lentils and lentil sprouts demonstrated a profound modification in the phenolic profiles of these products (Table 3). The retention time (*R_t_*), wavelengths of maximum absorption in the visible region (*λ_max_*), molecular ion, and fragment ion pattern allowed us to identify a total of twenty-two phenolic compounds, including hydroxybenzoic and hydroxycinnamic acids, catechins and procyanidins (flavan-3-ols), and flavonols and flavanones. Major free phenolic compounds in raw lentils were catechins and procyanidins (56%) (Figure 2, Table 3), whereas in the bound fraction, a high proportion of hydroxybenzoic acids (35%) was also present together with catechins (45%). The germination of lentils produced a significant loss of phenolic (hydroxybenzoic and hydroxycinnamic) acids (*p* < 0.01) and flavan-3-ols (*p* < 0.001) in the free phenolic fraction. In the bound phenolic fraction, the decreases were more remarkable for catechins (65% reduction, *p* < 0.001), leading to an increase in the proportion of hydroxybenzoic acids (1.4-fold increase). Concerning the load of total phenolics, the only differences were found for the proportion of flavonols and catechins; catechins decreased (from 55 to 41%) while flavonols increased (from 34 to 48%).

The main free phenolic compounds found in raw lentils were kaempferol dirutinoside, (+)-catechin 3-*O*-hexoside, and (+)-catechin (408.6, 298.6, and 263.0 μg/g, respectively) (Table 3). These compounds underwent a substantial reduction during germination (25, 31, and 43%, respectively), leading up to increases in the concentrations of other kaempferol derivatives. During germination, glucosidase activity is enhanced, resulting in a loss of the glycosidic forms of phenolic compounds [40]. β-Glycosidases, upregulated during germination, catalyzes the hydrolysis of β-glycosidic di- and other glycoside conjugates from phenolics, thus releasing both the sugar moiety and the aglycone [40]. Here, we observed an increase in the concentration of both kaempferol rutinoside rhamnoside isomers (2.2-fold) and primarily kaempferol rhamnoside (6.6-fold), produced from the elimination of one glucose or one rutinose (6-O-rhamnosil-glucose) molecules, respectively. Likewise, germination brought about a decrease in the concentration of procyanidin and prodelphinidin dimers, which was also observed by Lopez et al. [41]. Concerning bound phenolics, besides the losses in catechin due to germination, a slight decrease (33%, *p* < 0,05) was shown in the content of total flavonols, mainly due to the reduction in the concentration of kaempferol dirutinoside (*p* < 0.01) even though the content of kaempferol rhamnoside increased 1.8-fold (*p* < 0.0001). The content of total phenolics significantly diminished (33%, *p* < 0.001), following a similar trend to that observed for the TPC content. Besides that, the content of total phenolic compounds measured using HPLC-DAD-MS/MS strongly correlated (*r* = 0.9980, *p* < 0.001) with the values obtained using the colorimetric Folin-Ciocalteu method, validating the use of this spectrophotometric method as a screening technique for the analysis of phenolics during different processes [42].

### 3.2. Bioavailability of Melatonin from Lentil Sprouts and its Correlation with Plasma Antioxidant Capacity

**Figure 3:**
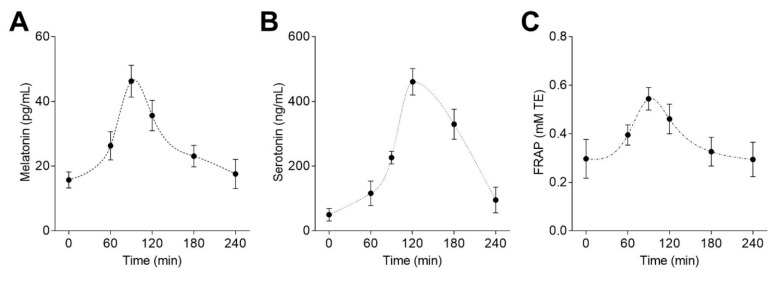
Plasma concentration-time profiles of melatonin (**A**), serotonin (**B**), and antioxidant capacity measured by the FRAP method (**C**) after the administration of lentil sprouts (equivalent to 50 µg/kg melatonin). Each point represents the mean ± SD (*n* = 8).

A pharmacokinetic study was performed using eight male rats after oral administration of lentil sprouts (50 µg melatonin/kg). Figure 3 shows the mean plasma concentration-time profiles of melatonin, serotonin, and the plasmatic antioxidant capacity measured by the FRAP method. Melatonin (Figure 3A) was detected in basal levels at time 0, following an increase in the concentration (45.4 pg/mL) until 1.5 h postadministration of the lentil sprouts extract. The concentration diminished from that point to achieve basal concentration 4 h after lentil sprouts intake. Regarding plasmatic serotonin concentration (Figure 3B), a significant increase was observed between 60−120 min after the consumption of lentil sprouts. Afterward, a pronounced decay in serotonin concentration was shown (28% at 180 min, 79% at 240 min). The antioxidant status of the plasma (Figure 3C) displayed similar behavior to melatonin. Increases in the antioxidant capacity were detected 60−90 min after the administration of lentil sprouts; then, a slight decrease was perceived before returning to the basal level after 4h. Melatonin concentration in the plasma significantly correlated with the antioxidant capacity (*r* = 0.989, *p* < 0.001). No significant (*p* > 0.05) correlation was detected with serotonin. Melatonin has been shown to reach maximum concentration between 20 to 100 min after its oral administration [43]; similar behavior was observed here. The enhancement of the plasmatic antioxidant capacity (measured by the FRAP method) seemed to be consistent with the antioxidant activity of melatonin. Melatonin was detected after the administration of synthetic melatonin (5 mg/kg) in Sprague-Dawley rats in much higher concentrations (1−2 µg/mL), showing that the co-ingestion with phenolic compounds (caffeic acid or quercetin) could increase melatonin bioavailability [44]. Therefore, the phenolic content (mainly represented by kaempferol glycosides, as previously observed in Table 3) present in the extract might not have a negative impact on melatonin bioavailability. Nevertheless, this hypothesis should be validated in future experiments.

Pharmacokinetic parameters were calculated from the plasmatic melatonin concentration–time profile (Table 4). After oral administration of lentil sprouts (50 µg/kg), melatonin displayed rapid absorption (*T_max_* = 90 min) followed by distribution and disposition in rat. The plasmatic half-life was around 100 min, indicating rapid metabolism. Elimination rate constant (*K_e_*) values were in the range of results previously obtained [44]. Indeed, melatonin mean residence time (MRT) was low (≈ 200 min). The low doses of melatonin (as such assayed in this study, 50 µg/kg) gave rise to lower MRT values. Thus, melatonin absorbed from foods (also low doses) only stays in the organism for short periods. The volume of distribution (*V_D_/F*) of melatonin was certainly high (0.85 L/g), implying a high distribution of the molecule into the tissues. The systemic plasma clearance (*Cl/F*) of melatonin was 329.9 L/h/kg, which was 100-fold the rat hepatic blood flow rate (3.3 L/h/kg). Melatonin is rapidly metabolized in the liver by cytochrome P450 enzymes, and then sulfated or glucuronidated. Melatonin can be degraded by nonenzymatic pathways involving radical species [45]. These results prove the rapid absorption and metabolism of melatonin from lentil sprouts after acute intake.

### 3.3. Lentil Sprouts Intake Greater Elevate Melatonin Biomarkers than the Synthetic Hormone

**Figure 4:**
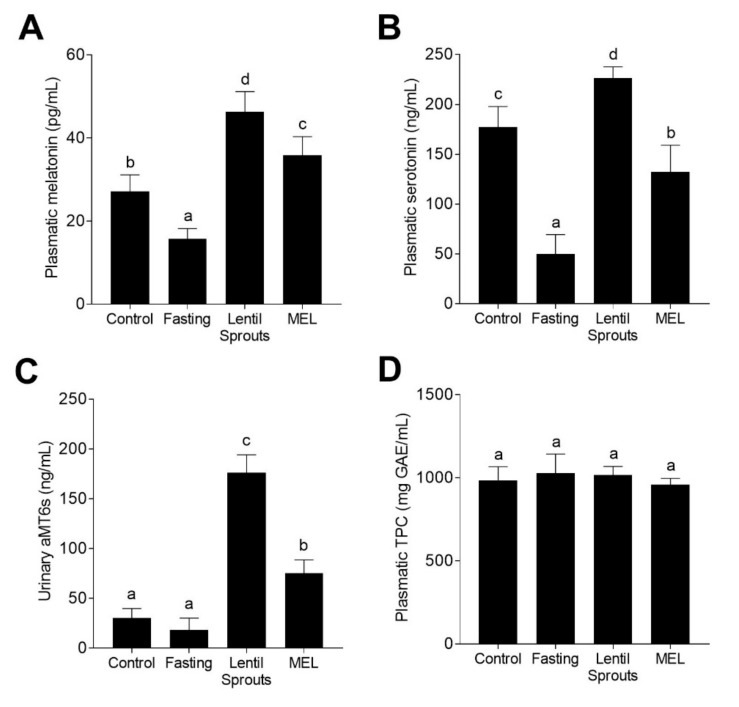
The concentration of melatonin (**A**), serotonin (**B**), 6-sulfatoxymelatonin (aMT6s) (**C**), and TPC (**D**) in the plasma and urine samples followed the four experimental conditions (Control, Fasting, Lentil Sprouts, and MEL). The results are expressed as mean ± SD (*n* = 10). Bars with different letters significantly (*p* < 0.05) differ according to ANOVA and Tukey’s multiple range test.

Based on the pharmacokinetic results, we compared plasmatic melatonin levels among four different feeding conditions (Control, Fasting, Lentil Sprouts, and MEL) 90 min after treatment (Figure 1). Plasmatic melatonin suffered a decrease (42%) when rats were subjected to a 12 h fasting period (Figure 4A). Periods of fast or caloric restriction have been associated with decreases in plasmatic melatonin concentrations [46]. Nevertheless, the literature shows discrepancies in the role of intermittent fasting and variations in the delivery of plasmatic melatonin [47]. The intake of lentil sprouts significantly (*p* < 0.05) increased melatonin concentration (1.7-fold in contrast to control; 2.9-fold in comparison to the Fasting group). The MEL group also exhibited a meaningful boost in melatonin plasmatic concentration, i.e., 22% lower than that of the Lentil Sprouts group. These outcomes extend the feasibility of consuming lentil sprouts as the melatonin food source. A significant part of the melatonin present in the diet can be absorbed by the gastrointestinal tract, thereby diminishing its levels in plasma. Numerous aspects may be associated with the plasmatic concentration of melatonin, including the age of the animals, the quantity of melatonin consumed, or the administration period [48]. Likewise, the intake of tryptophan has been associated with increased melatonin concentration in plasma [49]. Lentils are considered a source of tryptophan, both protein-bound and free [50]. Lentil sprout intake led to comparable increases of melatonin plasmatic concentration as those reported after walnut ingestion [51]. Fruit consumption can lead to increases in the plasmatic melatonin concentration [52], as can the intake of wine and beer [53]. The bioavailability of phytochemicals is distinctly influenced by the complexity of the food matrix, comprising plant cell-wall polysaccharides, dietary soluble, and insoluble fibers, among other components [54].

Additionally, the concentration of serotonin was studied (Figure 4B), observing a dramatic reduction when rats were fasted, whereas the treatment with the lentil sprout extract enhanced serotonin concentration (4.5-fold in contrast to the Fasting group) as well as the MEL group (2.7-fold). Since serotonin is a hormone related to digestion processes, the 12 h fasting period reduced (72%) the plasmatic concentration. Serotonin is mainly produced by enteroendocrine cells in the gastrointestinal tract in response to a variety of stimulants (nutrients, odorants, and phytochemicals, among others) [55]. Evidence shows that enterochromaffin cells secrete serotonin upon nutrient triggering [56,57]. Serotonin can, however, also be released as an adaptation to long-lasting fasting [58]. As formerly stated, the content of tryptophan from plant sources may also increase the production of extra-pineal melatonin through the metabolization via serotonin to melatonin. Both serotonin and melatonin are primarily synthesized (> 90%) by the gastrointestinal tract, from tryptophan and/or its derivatives, comprising a primary extrapineal pool of these molecules [59]. In fact, serotonin and melatonin can be synthesized not only by animal cells, but also by the microbiota present in the gastrointestinal tract [60].

Urinary aMT6s (Figure 4C), the main melatonin metabolite excreted in urine, did not suffer significant (*p* > 0.05) during the fasting period, while a substantial increase in its concentration was detected upon the administration of both lentil sprouts and melatonin solution (5.8- and 2.5-fold, respectively; *p* < 0.05). The concentration of this melatonin biomarker in urine was associated with the plasmatic concentration of melatonin (*r* = 0.926, *p* < 0.05). After intake, melatonin is promptly transformed in 6-hydroxymelatonin, being further conjugated, giving rise to aMT6s [61]. The concentration of aMT6s excreted in urine after lentil extract and melatonin intake confirmed the highly efficient metabolism of melatonin in rats [62]. The consumption of several fruits and vegetables previously evidenced the bioavailability of melatonin observed as an increase in the concentration of urinary aMT6s [52,63,64]. Nonetheless, the differences observed between Lentil Sprouts and MEL groups should be emphasized. Upon the intake of equal quantities of melatonin, the excretion of aMT6s was significantly (*p* < 0.05) different. Differences in the intake of tryptophan might be the cause of these different results. Tryptophan intake is associated with increased aMT6s production [65]. Tryptophan metabolization in the gastrointestinal tract could increase not only the synthesis of serotonin, but the final production of melatonin and the subsequent urinary excretion of aMT6s [66,67,68]. Hence, melatonin bioavailability depends not only on the initial concentration of melatonin in food and the matrix effect influencing its absorption, but also on the content of tryptophan which could be converted into melatonin and be part of the plasmatic melatonin load.

The concentration of TPC in the plasma of rats subjected to the four different treatments (Figure 4D) did not show any significant (*p* > 0.05) change in the 90 min posttreatment. The plasmatic *t_1/2_* of phenolic compounds frequently ranges 2 to 8 h, but it can reach up to 12–24 h for the larger phenolic structures [69]. In the present work, the levels of phenolic compounds in the plasma of 12 h-fasted rats did not show any fluctuations. The slow release of phenolics from food complex matrices such as regular rat chow, mainly based on vegetable food sources (wheat, corn, wheat bran, barley, soybean, among others) may be the cause of nonsignificant (*p* > 0.05) changes in the fasting group, as well as in the Lentil Sprouts or MEL groups [70]. The occurrence of phenolic compounds along the gut, due to their slow release from foods, appears to be the primary reason for the preservation of the total phenolic load in plasma [71]. Since gut microbiota mediates the synthesis of phenolic acids from larger phenolic compound polymers, such as proanthocyanidins, tannins, and glycosides, via ring fission and oxidation, and glycoside hydrolysis, the resulting phenolic metabolites can be absorbed and reach the systemic circulation [72,73]. Complex phenolic compounds and glycosides need to be metabolized in the colon to be absorbed. The main phenolic compounds composing the melatonin-rich extract administered, primarily kaempferol and (+)-catechin glycosides, may need these metabolization processes to reach their absorbable form and be bioavailable. Thus, the acute intake of lentil sprouts did not result in TPC modification, since phenolic compounds could not reach a digestion stage in which they were bioavailable.

### 3.4. Administration of Lentil Sprouts Enhances Plasmatic Antioxidant Status

**Figure 5:**
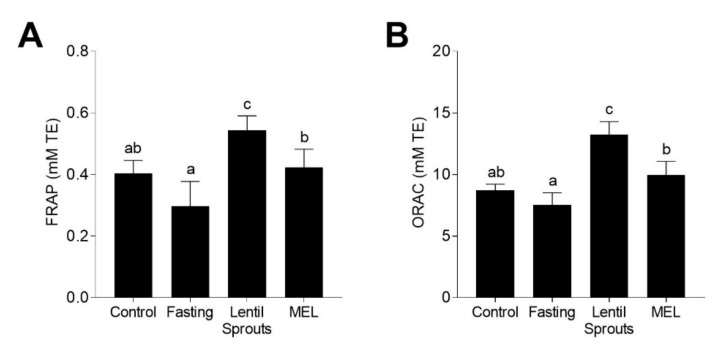
Plasmatic antioxidant capacity measured by FRAP (**A**) and ORAC (**B**) methods followed the four experimental conditions (Control, Fasting, Lentil Sprouts, and MEL). The results are expressed as mean ± SD (*n* = 10). Bars with different letters significantly (*p* < 0.05) differ according to ANOVA and Tukey’s multiple range test.

Since the major bioactivity of melatonin is its antioxidant potential, we measured the antioxidant status of the plasma by FRAP and ORAC methods 90 min after the treatments to establish the influence of lentil sprout intake (Figure 5). Even if the antioxidant capacity, measured by the FRAP method (Figure 5A), showed no significant (*p* > 0.05) changes during fasting, a 34% increase was observed in the Lentil Sprouts group and a 4% in MEL group. Similarly, the antioxidant capacity measured with the ORAC method showed a similar behavior (Figure 5B). Fasting did not cause significant (*p* > 0.05) alterations in the antioxidant capacity. However, the administration of lentil sprouts gave rise to a 1.8-fold increase; synthetic melatonin produced a less pronounced increment (32% in composition to the fasting group). The antioxidant capacity correlated with the concentration of melatonin (FRAP, *r* = 0.977, *p* < 0.05; ORAC, *r* = 0.962, *p* < 0.05) as well as with the concentration of urinary aMT6s (FRAP, *r* = 0.928, *p* < 0.05; ORAC, *r* = 0.991, *p* < 0.01). The antioxidant capacity did not display any significant correlation (*p* > 0.05) with the concentration of TPC in the plasma. Phenolics might have been slowly released, exhibiting different pharmacokinetics than melatonin. As previously observed, the intake of foodstuffs rich in melatonin such as fruits and derivates (cherries, grapes, and their juices), or fermented beverages (wines and beers) resulted in an enhancement of the plasmatic antioxidant capacity [25,26,53,74]. However, these results may involve a conservative evaluation of antioxidant capacity and should not be attributed solely to the presence of melatonin. These results could be related to other phytochemicals not measured, such as vitamin C or other antioxidants that could be associated with the increase in the plasmatic *in vitro* antioxidant capacity [75]. Furthermore, FRAP and ORAC assays exclusively asses the free radical scavenging capacity. Melatonin displays direct and indirect antioxidant properties, covering not only free radical scavenging capacity but the stimulation of endogenous antioxidant enzyme expression [76]. Melatonin administration has been correlated to the enhancement of the plasmatic antioxidant capacity in both animal models and human studies [77].

In general, melatonin intake is associated with diminished oxidative stress [78]. The supplementation with melatonin can be beneficial, beyond oxidative stress, in the reduction of inflammation, hypertension, and other metabolic syndrome-related markers [77]. Forthcoming studies will unravel the effects of lentil sprout intake on the gastrointestinal metabolism of melatonin and its influence on the oxidative status, both in plasma and in central tissues where oxidative stress is the primary cause of dysfunction, aging, and the development of chronic diseases. The synthesis of melatonin by the colonic microbiota presents alternative perspectives from which to understand how the content of melatonin increases after the intake of food. The gut microbiota could be considered as a supplementary source of extrapineal melatonin, produced by diverse types of bacteria, deserving future investigations [79].

## 4. Conclusions

In summary, the intake of lentil sprouts increased the concentration of melatonin in the plasma of rats as well as the urinary levels of 6-sulfatoxymelatonin. Likewise, the plasmatic antioxidant capacity was augmented. This study presents the composition of raw lentils and sprouts, and an evaluation of melatonin pharmacokinetics, bioavailability, and biological activity *in vivo* in Sprague Dawley rats. In view of the results, lentil sprouts can be considered as a source of bioavailable melatonin, whose intake could modulate plasmatic oxidative stress and defend the organism from aging and related diseases, among other potential health benefits. Future studies should be conducted to better understand the role of dietary melatonin and melatonin precursors in bioavailable melatonin biological actions and their influence on oxidative status, besides the potential use of lentil sprouts and other melatonin-rich foods to combat oxidative stress-related diseases.

## Figures and Tables

**Table 1:** Experimental conditions for the pharmacokinetic and bioavailability/bioactivity studies considering the intervention, rat population, date of entrance and time into inverse photoperiod, sampling day, mean group weight on the day of the experiment, and sampling time.

	Pharmacokinetic Study	Bioavailability and Bioactivity Study			
		Control	Fasting	Lentil Sprout	MEL
Group	1	2	3	4	5
Intervention	12 h-fast Lentil sprout	None	12 h-fast	12 h-fast Lentil sprout	12 h-fast Melatonin
Rat population (N)	8 ♂	10 ♂	10 ♂	10 ♂	10 ♂
Entrance into inverse photocycle (day)	23	21	24	29	29
Time in inverse photocycle (days)	22	25	20	20	21
Sampling day	45	46	44	49	50
Weight (g)	195 ± 11	203 ± 11	197 ± 9	198 ± 10	202 ± 8
Sampling time	10:00 to 14:00	11:00	11:00	11:00	11:00

**Table 2:** Melatonin content, free (FPC), bound (BPC), and total phenolic compounds (TPC), FPC: BPC ratio, and *in vitro* antioxidant capacity measured by the ORAC method in raw lentil and lentil sprouts and the equivalence of the dose of lentil sprouts given to rats.

	Melatonin (µg)	FPC (mg GAE)	BPC (mg GAE)	TPC (mg GAE)	FPC: BPC (ratio)	ORAC (µmol TE)
Raw lentils (per g)	(0.46 ± 0.06) · 10^‒3^	4.50 ± 0.17	0.38 ± 0.02	4.88 ± 0.19	(12 ± 1):1	20.24 ± 1.54
Lentil sprouts (per g)	1.01 ± 0.09 ***	3.15 ± 0.21 **	0.32 ± 0.03 *	3.47 ± 0.24 **	(10 ± 2):1	77.23 ± 5.78 ***
Dose (per kg)	50.0	155.9	51.0	171.8	-	52.0

Results are reported as mean ± SD (*n* = 3). Mean values followed by superscript asterisks significantly differ (raw vs. sprouted lentils) when subjected to *T*-test (^*^ *p* < 0.05, ^**^ *p* < 0.01, ^***^ *p* < 0.001)

**Table 3:** Retention time (*R_t_*), wavelengths of maximum absorption in the visible region (*λ_max_*), mass spectral data, tentative identification, and quantification (μg/g dry matter) of free and bound phenolic compounds present in raw lentils and lentil sprouts.

**Compounds**	*R_t_* **(min)**	***λ_max_*** **(nm)**	**Molecular ion [M-H]** ^−^ **(*m/z*)**	**MS^2^** **(*m/z*)**	**Concentration (μg/g)**			
					*Free phenolics*		*Bound phenolics*	
					Raw lentil	Lentil sprout	Raw lentil	Lentil sprout
***Hydroxybenzoic acids***								
***p*** **-Hydroxybenzoic-hexoside**	**5.48**	255	299	–	8.14 ± 4.16	3.34 ± 0.20	34.32 ± 6.84	27.06 ± 1.58
Protocatechuic acid	6.70	257, 294	153	–	23.22 ± 2.52	4.36 ± 0.60^***^	26.34 ± 3.00	21.48 ± 10.92
**Total**					**31.36 ± 6.68**	7.70 ± 0.80^**^	**60.66 ± 9.84**	**48.54 ± 12.50**
***Hydroxycinnamic acids***								
*trans-p*-Coumaric derivative	10.04	309	417	–	18.94 ± 2.08	5.16 ± 1.08^***^	nd	nd
*p*-Coumaroyl malic acid	12.46	308	278	163	9.50 ± 1.06	11.10 ± 2.88	nd	nd
*p*-Coumaroyl glycolic acid	12.80	314	220	163	12.48 ± 2.32	13.68 ± 2.16	nd	nd
*p*-Coumaric acid derivative	13.10	312	–	163	13.08 ± 0.78	nd^***^	nd	nd
*trans*-*p*-Coumaric acid	18.20	314	163	–	12.72 ± 0.00	8.78 ± 1.46^**^	2.58 ± 0.00	2.64 ± 0.62
*trans*-Ferulic acid	20.4	322	193	–	nd	nd	3.18 ± 0.34	3.16 ± 1.04
**Total**					**66.72 ± 6.24**	38.72 ± 7.58^**^	**5.76 ± 0.34**	**5.80 ± 1.66**
***Catechins and procyanidins***								
(+)-Catechin	10.90	280	289	245, 203, 161	262.98 ± 37.20	150.06 ± 14.08^**^	78.74 ± 14.54	27.94 ± 5.22^***^
(+)-Catechin 3-*O*-hexoside	9.02	280	451	289	298.58 ± 15.76	206.32 ± 5.26^***^	nd	nd
Dimer prodelphinidin	7.76	276	593	447, 441, 423, 305, 289	120.20 ± 2.54	nd^***^	nd	nd
Dimer procyanidin	10.40	279	577	289	52.68 ± 0.02	23.86 ± 2.00^***^	nd	nd
**Total**					**734.44 ± 55.52**	**380.24 ± 19.34** ^***^	**78.74 ± 14.54**	27.94 ± 5.22^***^
***Flavonols and flavanone***								
Kaempferol glucuronide dihexoside	10.12	346	785	285	nd	2.56 ± 0.60^**^	nd	nd
Kaempferol dirutinoside	14.05	346	901	755, 593, 285	408.60 ± 3.72	305.74 ± 10.04^***^	22.70 ± 4.72	8.44 ± 0.46^**^
Kaempferol rutinoside hexoside (I)	15.00	345	755	593, 285	15.02 ± 0.26	8.92 ± 1.26^**^	1.82 ± 0.12	1.90 ± 0.36
Kaempferol rutinoside hexoside (II)	17.04	346	755	593, 285	nd	7.10 ± 0.24^***^	nd	nd
Kaempferol rutinoside rhamnoside (I)	18.30	346	739	593, 285	12.68 ± 0.04	16.10 ± 1.00^**^	0.82 ± 0.06	1.24 ± 0.26
Kaempferol rutinoside rhamnoside (II)	19.14	346	739	593, 285	nd	11.32 ± 3.08^**^	nd	nd
Quercetin 3-glucoside	20.1	356	463	301	3.30 ± 0.26	3.02 ± 0.32	nd	nd
Kaempferol glucuronide	25.40	346	461	285	11.22 ± 2.48	14.10 ± 1.24	nd	nd
Kaempferol rhamnoside	34.10	346	431	285	10.52 ± 2.06	69.00 ± 8.54^***^	4.84 ± 0.46	8.76 ± 1.36^***^
Eriodyctyol hexoside	16.30	288, 338 (sh)	449	287	8.92 ± 1.26	18.78 ± 0.96^***^	nd	nd
**Total**					**470.26 ± 10.08**	**456.64 ± 27.28**	**30.18 ± 5.36**	20.34 ± 2.44^*^
***Total phenolic compounds***					***1302.78 ± 78.52***	***883.30 ± 55.00*** ^**^	***175.34 ± 30.08***	***102.62 ± 21.82*** ^*^

Results are reported as mean ± SD (*n* = 3). Mean values followed by superscript asterisks significantly differ (raw vs. sprouted lentils) when subjected to *T*-test (^*^ *p* < 0.05, ^**^ *p* < 0.01, ^***^ *p* < 0.001). nd: nondetected.

**Table 4:** Pharmacokinetic parameters of melatonin in rats after oral administration of lentil sprouts.

Parameter	Oral lentil sprouts (50 µg melatonin/kg)
*T_max_* (min)	90.0 ± 0.0
*C_max_* (pg/mL)	45.4 ± 3.5
*K_e_* (1/h)	0.39 ± 0.05
*t_1/2_* (min)	108.9 ± 13.0
AUC _0-t_ (pg/mL min)	6560 ± 963
AUC _0-t_ /dose (pg/mL min)/(μg/kg)	131.2 ± 19.3
AUC _0-∞_ (pg/mL min)	9381 ± 2008
AUC _0-t_ /AUC _0-∞_	0.71 ± 0.05
AUMC _0-∞_ (pg/mL min^2^)	1897356 ± 589030
MRT _0-∞_ (min)	199.4 ± 19.8
*V_D_/F* (μg/kg)/(pg/mL)	0.85 ± 0.08
*Cl/F* (μg/kg)/(pg/mL)/h	0.33 ± 0.07

*T_max_*: time to the maximal plasmatic concentration; *C_max_*: maximal plasmatic concentration; *K_e_: elimination rate constant; t_1/2_*: elimination half-life; MRT: mean residence time; *V_D_/F*: volume of distribution; *Cl/F:* clearance
